# Complete chloroplast genome of a high-quality forage in north China, *Medicago ruthenica* (Fabaceae：Trifolieae)

**DOI:** 10.1080/23802359.2020.1845578

**Published:** 2021-01-05

**Authors:** Jiuxiang Xie, Junping Mao, Zongren Li, Chengbo Liang, Weiyou Ou, Junwei Tang, Hua Liu, Haichun Wang, Zhongke Ji, Kazhuocairang, Yingfang Shen

**Affiliations:** a State Key Laboratory of Plateau Ecology and Agriculture, College of Agriculture and Animal Husbandry, Qinghai University, Xining, China; b Bureau of Natural Resources of Guinan County, Mangqu, China; c Qinghai Provincial General Grassland Station, Xining, China; d Forest station of Youganning Town, Henan Mongolian Autonomous County, Youganning, China; e Grassland Comprehensive Professional Team of Henan Mongolian Autonomous County, Youganning, China; f College of Ecological Environment and Resources, Qinghai Nationalities University, Xining, Qinghai, China

**Keywords:** *Medicago ruthenica*, chloroplast genome, phylogenetic analysis, Fabaceae

## Abstract

*Medicago ruthenica* is a well-known high-quality forage due to its good palatability and strong tolerance to drought, cold and saline-alkali stress. Here, the complete chloroplast genome sequence of *M. ruthenica* was reported. The chloroplast genome is 126,939 bp in length. This chloroplast genome has no inverted repeat (IR) regions, which is very common in the family Fabaceae. The *M. ruthenica* chloroplast genome encodes 107 genes, including 73 protein-coding genes, 30 tRNA genes, and 4 rRNA genes. Phylogenetic analysis result strongly suggested that *M. ruthenica* is a distinct lineage in *Medicago*, being sister to highly supported clade composed of three species (*M. hybrida, M. papillosa and M. sativa*).

*Medicago ruthenica* is a perennial plant, which is one of the core species of Section *Pialcarpae* (Small and Jomphe 1989). It is considered to be a relic species of Tertiary flora in the Palearctic (Campbell et al. 1997). It is widely distributed in Mongolia, Korea, Russia (Siberia, Far East) and high latitude and cold regions in northern China (Hao and Shi 2006). Its habitat is mostly in extremely cold areas and saline-alkali lands with little rain, little snow or no snow cover in winter (Balabaev 1934). It has strong tolerance to drought, cold and saline-alkali stress (Balabaev 1934). At the same time, *M. ruthenica* is a perennial wild legume species, has high-crude protein content and good palatability (Huang, et al., 2007). For these reasons, *M. ruthenica* is considered to be a high-quality perennial leguminous forage resource with domestication potential, which is expected to be cultivated and utilized in areas where *M. sativa* and other alfalfa species cannot survive the winter. However, no studies on the plastome of *M. ruthenica* have been published. In this study, the complete chloroplast genome of *M. ruthenica* (Genbank accession number: MN901635) was sequenced on the Illumina NavoSeq Platform (Illumina, San Diego, CA, USA), which will provide genetic and genomic information to promote its ecological restoration and systematics research of Fabaceae.

In this study, *M. ruthenica* were collected from Mari village, Xunhua County, Haidong City, Qinghai Province, China (35.68°N, 102.35°E). The fresh and young leaves were dried immediately by silica gels. The complete chloroplast genome of *M. ruthenica* was extracted from the dried leaves (about 0.2 g) with a modified CTAB method. The voucher specimen was kept in Herbarium of the Northwest Institute of Plateau Biology, Northwest Institute of Plateau Biology, Chinese Academy of Sciences (HNWP, XIE2019015). Genome sequencing was performed using the Illumina NovaSeq Platform at Genepioneer Biotechnologies Inc., Nanjing, China. The trimmed reads were mainly assembled by SPAdes v3.10.1 (Bankevich et al. 2012). Then PCGs, rRNAs and tRNAs were annotated by prodigal v2.6.3, hmmer v3.1b2 and aragorn v1.2.38, respectively.

The complete chloroplast genome of *M. ruthenica* has an atypical chloroplast genome structure with a length of 126,939 bp. This chloroplast genome has no inverted repeat (IR) regions, which is very common in the family Fabaceae. The GC content of the whole chloroplast genome is 34.12%. A total of 107 functional genes were annotated, including 73 protein-coding genes, 30 tRNA genes, and 4 rRNA genes. Fourteen of them contain one intron and 1 of them contains two introns.

Based on the complete chloroplast genomes assembled here and downloaded from GenBank, phylogenetic relationships of 8 Fabaceae species (7 and 1 from Medicago and Lathyrus, respectively) with two species from Crassulaceae as outgroups were resolved by the mean of maximum likelihood with 1000 bootstrap repeats (model: F81 + F + I + G4). After aligned using MAFFT (Katoh and Standley 2013), the maximum likelihood tree (Figure 1) was built using W-IQ-TREE (Trifinopoulos et al. 2016). The phylogenetic tree showed that *M. ruthenica* was a distinct lineage in *Medicago*.

**Figure 1.**
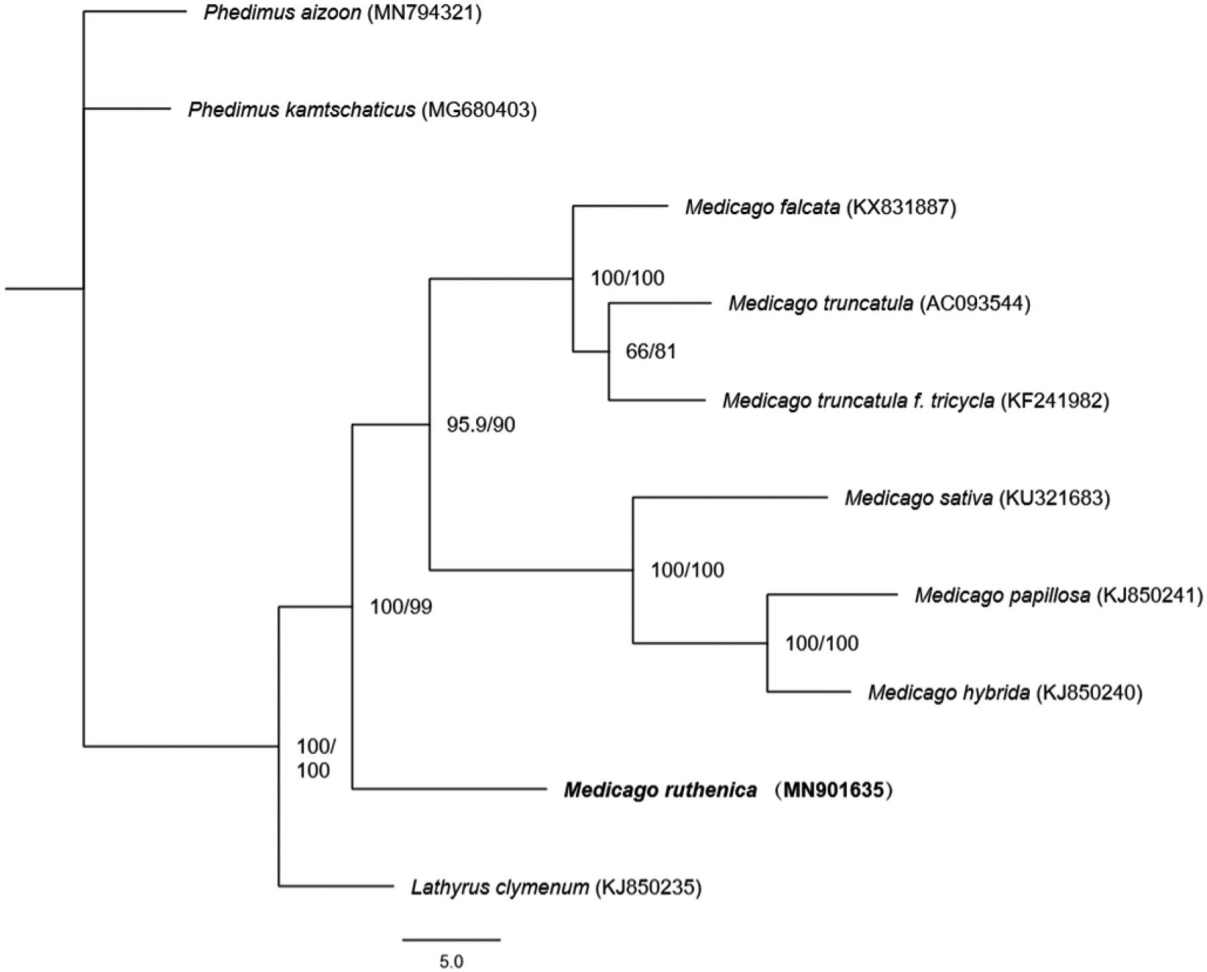
The maximum likelihood tree based on 10 complete chloroplast genome sequences. Support values written on the branches: SH-aLRT support (%)/ultrafast bootstrap support (%).

## Data Availability

The data that support the findings of this study are openly available in GenBank of National Center for Biotechnology Information at https://www.ncbi.nlm.nih.gov, reference number MN901635.
